# Are cytokines (IL-6, CRP and adiponectin) associated with bone mineral density in a young adult birth cohort?

**DOI:** 10.1186/s12891-018-2357-3

**Published:** 2018-11-30

**Authors:** Ana Maria Baptista Menezes, Paula Duarte Oliveira, Helen Gonçalves, Isabel O. Oliveira, Maria Cecilia F. Assunção, Luciana Tovo-Rodrigues, Gustavo Dias Ferreira, Fernando César Wehrmeister

**Affiliations:** 1 Federal University of Pelotas – Post-Graduate Program in Epidemiology, Rua Marechal Deodoro, 1160, 3° andar, Pelotas, RS Zip code: 96020-220 Brazil; 2 Department of Physiology and Pharmacology Campus Capão do Leão, Federal University of Pelotas, Pelotas, RS Zip code: 96010-900 Brazil

**Keywords:** Cytokines, Interleukin-6, C-reactive protein, Adiponectin, Inflammatory markers, Anti-inflammatory markers, Bone mineral density (BMD), Cohort studies

## Abstract

**Background:**

Studies have shown that cytokines play a role in bone remodeling.

**Methods:**

In 1993, all hospital births occurred in Pelotas (Brazil) were identified and a total of 5249 newborns were included in the present cohort. Sub-samples of this cohort were visited during childhood and all members were traced at 11, 15, 18 and 22 years old. At 18 and 22 years the following biomarkers were measured: IL-6, CRP and adiponectin (the last one in a sub-sample) and bone mineral density (BMD-mg/cm^2^) was evaluated at 22 years. Crude regression analysis as well as adjusted for confounders (birth weight, pregnancy maternal smoking, gestational age, skin color, schooling, income, smoking, alcohol, physical activity, medical diagnosis of asthma, diabetes and hypertension, BMI, height, calcium intake, corticosteroid use, age at menarche, insulin and testosterone) were performed between the three biomarkers and the whole-body, lumbar spine and femoral BMD.

**Results:**

No statistical significant association was found between IL-6 and CRP with BMD, in males. Significant inverse association in the adjusted analysis, among females, was found for the highest tertiles of CRP at 22 y (beta − 15.2 mg/cm^2^; 95% CI: -25.4; − 4.9; *p* = 004), of CRP and IL-6 at 22 years (beta − 20.0 mg/cm^2^; 95% CI: -31.7; − 8.3; *p* = 0.003), and of IL-6 and CRP at both ages (beta − 20.3 mg/cm^2^; 95% CI: -38.0; − 2.5; *p* = 0.001) with total body BMD. Significant association, among males, was also found between the highest tertile of adiponectin at 22 y (beta − 23.3 mg/cm^2^; 95% CI: -35.5; − 11.1; *p* = < 001; beta − 22.5 mg/cm^2^; 95% CI: -42.9; − 2.2; *p* = 0.03; and beta − 31.8 mg/cm^2^; 95% CI: -55.5; − 9.1; *p* = 0.006) and total body, lumbar spine and femur neck BMD, respectively; and, among females, − 17.8 mg/cm^2^; 95% CI: -34.9; − 0.9; *p* = 0.033, with lumbar spine BMD.

**Conclusion:**

CRP at 22 years, in females, seems to be a marker for total body BMD; adiponectin at 22 years is also a marker for BMD at the three sites, in males, and for lumbar spine BMD, in females.

**Electronic supplementary material:**

The online version of this article (10.1186/s12891-018-2357-3) contains supplementary material, which is available to authorized users.

## Background

Peak bone mass, which is attained in young adulthood, is an important predictor of bone mineral density (BMD) late in life [1, 2]. Also, there is evidence in the literature that lower bone mass values in adulthood are associated with higher chance of low-energy osteoporotic fractures [3, 4].

Inflammation seems to be one of the determinants of bone fragility, being optimal control of inflammation part of the prevention of osteoporosis. However, this relationship is still under study, and the potential role of inflammatory markers modulating the effects on bone can help us to identify early measures for preventing future bone diseases.

Among the various cytokines affecting bone metabolism, interleukin-6 (IL-6), C-reactive protein (CRP) and adiponectin can play an important role [5–7] in the activation of osteoclasts, but studies on this subject are still ongoing, mainly at young adulthood and with longitudinal design.

The relationship between cytokines and BMD should be evaluated considering potential confounders, such as obesity, since a positive IL-6 and CRP association and a negative adiponectin and adiposity association have been demonstrated [8–10]. Other variables may also be responsible for this association such as health behaviors (physical activity, smoking and drinking), socioeconomic status, morbidities, diet and hormonal levels; in addition to these exposures, it is known that smoking during pregnancy, gestational age and birth weight can also influence bone health during life [11].

The aim of this study was to identify whether there was an association of cytokines at 18 and 22 years old with bone mineral density at 22 years old, shedding some light in the understanding of osteoporosis and consequent susceptibility to fractures in older ages.

## Methods

All livebirths occurred in the five hospitals in the city of Pelotas, Brazil, in the 1993 calendar year, were eligible to participate in a birth cohort study. A total of 5249 newborns were included in the study (16 refusals) and we were able to interview the mothers about their pregnancy and to carry out anthropometric measurements of the babies; more detailed information about the perinatal phase of the study have been published previously [12]. Sub-samples of this cohort were visited during childhood and all members of the cohort were traced at 11, 15, 18 and 22 years old with a response rate of 81.4 and 76.3% including the deaths, in the last two visits, respectively [13, 14].

Bone mineral density (BMD) at 22 years was measured in milligrams per square centimeter (mg/cm^2^), at three sites (total body, lumbar spine and femoral neck) by dual-energy X-ray absorptiometry (DXA) using a Lunar Prodigy Advance Bone Densitometer (GE, Germany). Every morning, before measurements, daily quality assurance procedure was completed and if the system did not pass the test, the quality assurance procedure was done again. Two trained technicians were responsible for the exams, with participants in supine position using light and tight-fitting shorts and sleeveless tops. DXA scans were not performed in wheelchair users and/or individuals with osteoarticular deformities, extremely obese individuals, or those with height over1.92 m; participants should remove all metal accessories such as bracelets, earrings or piercings. The examiners assessed the quality of DXA exams with participants still on the machine and repeated it if necessary; BMD was analyzed as a continuous variable.

At the age of 18 and 22 years, non-fasting blood samples were drawn by venipuncture using vacutainer tubes and samples were processed and stored in ultra-low temperature freezers in a central biorepository. Three cytokines were evaluated and considered as the main exposures: a) IL-6 (pg/mL) analyzed by the Quantikine® HS Human IL-6 immunoassay kit (R&D Systems®, Inc.; Minneapolis, MN55413, USA); b) CRP (mg/L) by immunoturdimetric assay (Labtest Diagnóstica SA, Minas Gerais, Brazil); and c) Adiponectin (μg/mL) assayed with the ELISA Quantikine Human Total Adiponectin Immunoassay kit (R&D Systems, Inc., Minneapolis, USA); at the age of 18 years, adiponectin was measured in a small random sample because the lack of funding for carrying out the analysis among all the members of the cohort.

The intra and inter assay precision of the tests were: for adiponectin (9.11–13.18%), IL-6 (4.1–13.37%) and for hsCRP (1.98–2.09%), respectively.

Due to the observed non-linear relationship in some of the fractional polynomials models applied to test the association between the main exposures and BMD, we opted for the tertiles categorization of the exposures; however, we carried out the same analysis using linear regression using the continuous variables excluding outliers (values out of ±2 z-score range - log scale for CRP and IL-6) (see Additional file 1: Table S1 and Additional file 2: Figures. S1 to S6). The cytokines were categorized as follow: a) CRP and IL-6 in tertiles at 18 and 22 years; b) CRP and IL-6 in the highest tertile, classified as none, only CRP or IL-6, and both CRP and IL-6 in each age; c) CRP or/and IL-6 in the highest tertile, combining the two follow-ups, classified as none, only at 18, only at 22, and both 18 and 22 years. Adiponectin was classified in the same way, except for the exposure of combined cytokines; the results were reported in a separate table due to the anti-inflammatory role of adiponectin compared to the inflammatory role of IL-6 and CRP.

The exclusion criteria for the blood sample and for DXA were refusal or pregnancy in women.

The covariates taken into account in the present analysis were: a) collected at birth: gestational age (weeks) estimated from the last menstrual period; smoking during pregnancy as a dichotomous variable (smoker/non-smoker); maternal skin color (white/black/brown/others); and birth weight (measured by hospital staff with 10-g precision pediatric scales calibrated regularly by the research team); b) collected at 18 and 22-years follow-ups: age at menarche for females (collected at the 18 years visit), smoking (no/yes for smoking at least one time per week), harmful alcohol intake (Alcohol Use Disorders Identification Test – AUDIT) [15], physical activity (minutes/week - measured through standardized and previously tested International Physical Activity Questionnaire - IPAQ) [16], daily calcium intake (mg adjusted by total calories consumption - measured through Food Frequency Questionnaire) [17, 18] and, collected at 18 and 22 years, but only 22-years follow-up information was included in the analyses: schooling (successfully complete years); asset index (quintiles); body mass index (BMI - kg/m^2^); medical diagnosis at any time during life referred by the participants (yes/no for hypertension, asthma and/or diabetes), and insulin (μU/mL) and testosterone (ng/d) blood sample (the last two measurements available only for the age of 22 years).

Descriptive analyses were performed using absolute and relative frequencies for categorical variables and mean and standard deviations (SD) for continuous variables. Association of all cytokines according to tertiles at the different ages of follow-ups and BMD at all sites was tested by unadjusted and adjusted linear regression analysis and reported as β coefficients and its 95% confidence intervals (95% CI). We tested the interaction between the main exposures and sex including an interaction term in the regression models; since most of the results achieved a *p* value lower than 0.05, analysis was stratified according to sex. *P*-values were obtained by Wald’s test for linear tendency or Wald’s test for heterogeneity, as appropriate. Variance inflation factor test was performed after regressions to ensure the absence of multicollinearity in the adjusted models.

All analysis was performed using Stata 12 software (StataCorp, College Station, Texas).

All the cohort follow-up projects were approved by the Research Ethics Committee of the Federal University of Pelotas Medical School after 1996 (ethical approval for studies was not required in Brazil until the referred year). At the 18 and 22 follow-ups, the projects were approved under the protocols 05/11 and 1.250.366, respectively.

All participants, or their parents or caregivers in the follow-ups before the participants reached 18 years, signed a written consent form in each follow-up and dataset was anonymized for the analyses.

## Results

The sample was composed by 3523 subjects (52.8% females), who had complete information on IL-6 and CRP and bone mineral density at the age of 18 and 22 follow-ups; for adiponectin, the total sample was 1706, due to the very small sample size with measured adiponectin at 18 years. Perinatal information (Table 1) shows 9.3% of low birthweight, 10.3% of prematurity and one third of maternal smoking during pregnancy. Whereas the percentage of current smoking, physical inactivity and obesity (BMI ≥ 30 kg/m^2^) increased from 18 to 22 years around one quarter, 40 % and nearly twice, respectively, there was a reduction of around 16% for harmful alcohol intake in the period. Medical diagnosis of hypertension, diabetes and asthma was slightly more frequent at the 22 years than at 18 years; at the last visit, almost 3% referred to use corticosteroid in the last 3 months, and oral contraceptive, among females, was reported by 60%; around 11% of the women were currently breastfeeding and 52% reported the age at menarche from 12 to 13 years old (Table 1).

**Table 1:** Sample description according to categorical covariates

	Perinatal	
Birth weight		
< 2500 g	291 (9.3)	
≥ 2500 g	2854 (90.7)	
Gestational age		
< 37 weeks	322 (10.3)	
≥ 37 weeks	2819 (89.7)	
Maternal smoking during pregnancy	1138 (32.3)	
Skin color		
White	2168 (63.6)	
Black	515 (15.1)	
Brown	598 (17.5)	
Others	128 (3.8)	
	18 years N (%)	22 years N (%)
Schooling (complete years)		
0–4	155 (4.4)	89 (2.5)
5–8	1376 (39.1)	945 (26.9)
9–11	1846 (52.4)	1453 (41.3)
≥ 12	144 (4.1)	1032 (29.3)
Asset index (quintiles)		
1st	703 (20.0)	697 (19.8)
2nd	687 (19.5)	708 (20.1)
3rd	703 (20.0)	701 (20.0)
4th	718 (20.4)	704 (20.0)
5th	709 (20.1)	708 (20.1)
Current smoking	464 (13.2)	576 (16.4)
Harmful alcohol intake (AUDIT ≥ 8 points)	902 (25.6)	752 (21.4)
Physical inactivity^a^	1398 (39.8)	1966 (56.0)
BMI (kg/m^2^)		
< 25	2488 (72.5)	1890 (56.7)
25–29.9	647 (18.9)	904 (27.1)
≥ 30	295 (8.6)	538 (16.2)
Medical diagnosis		
Hypertension	299 (8.5)	326 (9.3)
Diabetes	131 (3.7)	147 (4.2)
Asthma	750 (21.3)	805 (22.9)
Corticoids use in the last three months	73 (2.2)	98 (2.9)
Women variables		
Oral contraceptive use	1141 (61.3)	1098 (59.0)
Currently breastfeeding	83 (4.5)	202 (10.9)
Age at menarche (years)^b^		
≤ 11	516 (27.9)	
12–13	962 (52.0)	
≥ 14	372 (20.1)	

Individuals followed at 18 and 22 years (*n* = 3523) ^a^Classified as inactive those who do not reach 300 and 150 min/week of physical activities in leisure and commuting, at 18 and 22 years, respectively. ^b^Information collected at 18 years interview. AUDIT: Alcohol Use Disorder Identification Test

The mean (SD) of the anthropometric variables, cytokines (IL-6, CRP and adiponectin) and bone mineral density (BMD) at three sites (whole-body, lumbar spine and femoral neck) are shown in Table 2, stratified by sex.

**Table 2:** Sample description according exposures, outcomes and continuous covariates

	Males (*n* = 1663) Mean (SD)		Females (*n* = 1860) Mean (SD)	
	18 years	22 years	18 years	22 years
Height(cm)	173.9 (7.0)	174.4 (7.1)	161.1 (6.4)	161.1 (6.5)
BMI (kg/m^2^)	23.3 (4.1)	25.0 (4.8)	23.5 (4.8)	25.4 (5.8)
Daily calcium intake (mg)	717.6 (334.9)	737.6 (320.7	692.3 (358.4)	770.3 (328.4)
Testosterone (ng/dL)	–	517.1 (183.5)	–	23.9 (31.4)
Insulin (μU/mL)	–	26.3 (24.8)	–	33.9 (33.3)
IL-6 (pg/mL)	1.6 (2.0)	1.6 (1.8)	1.8 (1.9)	1.8 (1.8)
IL-6 (log pg/mL)	0.2 (0.6)	0.2 (0.7)	0.3 (0.7)	0.3 (0.7)
CRP (mg/L)	1.5 (3.0)	1.8 (4.9)	3.2 (4.6)	3.7 (7.9)
CRP (log mg/L)	−0.4 (1.2)	−0.3 (1.2)	0.3 (1.3)	0.5 (1.3)
Adiponectin (μg/mL)	14.3 (7.0)	7.9 (3.9)	17.0 (7.9)	10.6 (4.7)
BMD (mg/cm^2^)				
Total body	1225.3 (94.5)	1270.2 (97.1)	1134.1 (77.8)	1157.9 (80.3)
Lumbar spine	1178.1 (132.9)	1236.3 (141.9)	1158.2 (125.2)	1200.6 (129.3)
Femoral neck	1188.5 (157.4)	1178.4 (166.3)	1034.8 (127.8)	1028.6 (129.3)

*BMI* body mass index, *IL-6* interleukin-6, *CRP* C-reactive protein, *BMD* bone mineral density

Insulin and testosterone information available only at 22 years

Tables 3 and 4 show the unadjusted and adjusted association between IL-6 and CRP measured at both visits with whole-body, lumbar spine and femoral neck BMD, in males and females, respectively, measured at 22 y. Due to the change of direction in some of the adjusted regression coefficients compared to the unadjusted coefficients (from direct coefficients in the crude to inverse coefficients in the adjusted analysis), only the adjusted results will be mentioned in the text. For males (Table 3), no statistical significance was found for the association between cytokines (IL-6 and CRP) at 18 or 22 years and BMD at the different sites. For females (Table 4), there was no statistical difference for the association between IL-6 and CRP at 18 years with BMD, and the majority of the regression coefficients in the adjusted analysis did not show a change of direction. Nevertheless, CRP in the highest tertile at 22 years, and both cytokines in the highest tertile analyzed together, showed an inverse and statistical significant association with total body BMD, respectively (− 15.2 mg/cm^2^ 95% CI: -25.4; − 4.9; *p* = 0.004 and − 20.0 mg/cm^2^ 95% CI: -31.7; − 8.3; *p* = 0.003). CRP in the highest tertile at 18 and 22 y also showed an inverse statistical significant association with total body BMD (*p* = 0.002). For both cytokines in the highest tertile at both ages we found an inverse beta of − 20.3 mg/cm^2^ (95% CI: -38.0; − 2.5) with a *p* = 0.001;

**Table 3:** CRP and Il-6 at 18 and 22 years old and BMD at 22 years, males

	BMD - 22 years (mg/cm^2^)					
	Total body β (95% CI)		Lumbar spine β (95% CI)		Femur neck β (95% CI)	
	Crude	Adjusted	Crude	Adjusted	Crude	Adjusted
18 years						
CRP (log mg/L - tertiles)	*p = 0.009*	*p = 0.160*	*p = 0.280*	*p = 0.082*	*p = 0.320*	*p = 0.194*
1	Ref.	Ref.	Ref.	Ref.	Ref.	Ref.
2	12.9 (0.4; 25.4)	−5.2 (−17.1; 6.7)	8.6 (−9.6; 26.9)	− 10.2 (−29.9; 9.6)	11.4 (− 10.0; 32.7)	−10.3 (−32.3; 11.8)
3	15.3 (3.5; 27.1)	−8.3 (− 20.0; 3.4)	9.1 (− 8.1; 26.3)	− 17.0 (− 36.4; 2.4)	9.6 (− 10.6; 29.8)	−14.0 (− 35.7; 7.6)
IL-6 (log pg/mL - tertiles)	*p = 0.625*	*p = 0.90*	*p = 0.596*	*p = 0.317*	*p = 0.882*	*p = 0.504*
1	Ref.	Ref.	Ref.	Ref.	Ref.	Ref.
2	−8.9 (−21.3; 3.5)	−17.2 (− 29,0; − 5.4)	− 7.0 (− 25.0; 11.0)	−16.1 (− 35.7; 3.4)	−4.0 (− 25.0; 17.0)	−8.7 (− 30.5; 13.1)
3	−3.0 (− 15.3; 9.4)	− 10.3 (− 22.1; 1.6)	−4.8 (− 22.7; 13.2)	−10.0 (− 29.6; 9.7)	1.7 (− 19.3; 22.7)	−7.4 (− 29.4; 14.5)
CRP and IL-6 in the highest tertile	*p = 0.264*	*p = 0.364*	*p = 0.830*	*p = 0.308*	*p = 0.672*	*p = 0.415*
None	Ref.	Ref.	Ref.	Ref.	Ref.	Ref.
Only CRP or IL-6	11.4 (− 0.4; 23.1)	4.2 (−7.2; 15.5)	5.8 (− 11.3; 23.0)	−6.8 (− 25.6; 12.0)	11.3 (− 8.8; 31.3)	− 3.2 (− 24.2; 17.8)
Both CRP and IL-6	4.3 (−9.5; 18.2)	−8.6 (− 21.8; 4.5)	0.1 (− 20.0; 20.2)	− 10.3 (− 32.1; 11.4)	1.10 (− 22.5; 24.7)	− 10.4 (− 34.8; 14.0)
22 years						
CRP (log mg/L - tertiles)	*p = 0.001*	*p = 0.939*	*p = 0.032*	*p = 0.413*	*p = 0.008*	*p = 0.834*
1	Ref.	Ref.	Ref.	Ref.	Ref.	Ref.
2	21.4 (9.1; 33.7	−0.3 (−12.2; 11.6)	16.0 (−1.9; 33.8)	−3.0 (− 22.7; 16.7)	18.6 (− 2.2; 39.5)	−5.6 (− 27.6; 16.5)
3	20.5 (8.6; 32.5)	0.5 (− 11.6; 12.6)	18.7 (1.3; 36.2)	8.5 (− 11.3; 28.6)	27.0 (6.7; 47.3)	3.8 (− 18.5; 26.1)
IL-6 (log pg/mL - tertiles)	*p = 0.018*	*p = 0.250*	*p = 0.275*	*p = 0.193*	*p = 0.072*	*p = 0.216*
1	Ref.	Ref.	Ref.	Ref.	Ref.	Ref.
2	7.9 (−4.4; 20.1)	4.5 (−7.4; 16.3)	12.8 (−5.0; 30.7)	11.5 (−8.1; 31.1)	9.3 (−11.5; 30.2)	5.7 (−16.1; 27.6)
3	15.0 (2.6; 27.5)	7.2 (−5.1; 19.6)	9.8 (−8.3; 28.0)	13.4 (−7.0; 33.8)	19.4 (−1.8; 40.5)	14.4 (−8.4; 37.2)
CRP and IL-6 in the highest tertile	*p = 0.014*	*p = 0.527*	*p = 0.211*	*p = 0.225*	*p = 0.025*	*p = 0.258*
None	Ref.	Ref.	Ref.	Ref.	Ref.	Ref.
Only CRP or IL-6	5.1 (−6.8; 17.1)	−1.4 (−13.0; 10.1)	4.9 (−12.6; 22.4)	4.3 (− 14.9; 23.4)	14.5 (−5.9; 34.8)	5.1 (− 16.3; 26.4)
Both CRP and IL-6	17.6 (4.0; 31.2)	5.6 (−8.1; 19.3)	12.58 (−7.3; 32.4)	14.4 (− 8.2; 37.1)	24.7 (1.6; 47.8)	14.8 (−10.5; 40.1)
18 and 22 years^a^						
CRP in the highest tertile	*p = 0.065*	*p = 0.469*	*p = 0.279*	*p = 0.155*	*p = 0.071*	*p = 0.378*
None	Ref.	Ref.	Ref.	Ref.	Ref.	Ref.
Only at 18	12.2 (−2.1; 26.5)	−1.8 (15.6; 12.1)	9.1 (−11.7; 30.0)	−7.6 (−30.5; 15.3)	11.1 (− 13.3; 35.5)	− 2.1 (− 27.8; 23.6)
Only at 22	15.2 (1.3; 29.1)	6.2 (−7.4; 19.8)	19.4 (−0.9; 39.7)	20.5 (−2.0; 43.0)	31.8 (8.1; 55.5)	17.9 (−7.2; 43.0)
Both 18 and 22 years	13.9 (− 1.1; 29.0)	−7.5 (−22.3; 7.2)	10.1 (−11.9; 32.1)	−6.9 (− 31.4;17.5)	11.6 (−14.0; 37.1)	−8.2 (− 35.5; 19.0)
IL-6 in the highest tertile	*p = 0.258*	*p = 0.708*	*p = 0.935*	*p = 0.851*	*p = 0.365*	*p = 0.681*
None	Ref.	Ref.	Ref.	Ref.	Ref.	Ref.
Only at 18	−.19 (−13.8; 13.4)	0.5 (−12.5; 13.4)	−4.7 (−24.6; 15.3)	−3.3 (−24.7; 18.1)	− 4.49 (−27.7; 18.8)	−4.6 (−28.6; 19.3)
Only at 22	11.4 (−2.6; 25.4)	7.9 (−5.6; 21.4)	1.3 (−19.0; 21.7)	7.5 (− 14.8; 29.9)	8.42 (−15.3; 32.1)	12.0 (− 13.0; 36.9)
Both 18 and 22 years	10.7 (− 4.8; 26.3)	1.1 (− 14.0; 16.2)	3.5 (− 19.2; 26.2)	5.0 (−20.1; 30.0)	20.4 (− 6.0; 46.8)	7.6 (−20.3; 35.5)
Both CRP and IL-6 in the highest tertile	*p = 0.117*	*p =* 0.268	*p = 0.692*	*p = 0.505*	*p = 0.336*	*p = 0.561*
None	Ref.	Ref.	Ref.	Ref.	Ref.	Ref.
Only at 18	−0.9 (− 16.8; 15.0)	−11.3 (− 26.4; 3.9)	−2.95 (− 26.2; 20.3)	−9.8 (−35.0; 15.3)	−5.7 (− 33.0; 21.5)	− 12.4 (− 40.7; 15.8)
Only at 22	17.7 (2.2; 33.2)	8.5 (−6.9; 24.0)	12.8 (− 9.8; 35.5)	15.7 (− 10.0; 41.3)	21.8 (−4.6; 48.2)	14.7 (− 14.0; 43.3)
18 and 22 years	11.2 (−10.5; 32.9)	−3.5 (−23.9; 16.9)	4.7 (− 26.7; 36.2)	−1.8 (−31.7; 35.4)	12.8 (− 23.9; 49.5)	3.4 (−34.1; 40.9)

Total males sample *n* = 1486

*IL-6* interleukin-6, *CRP* C-reactive protein, *BMD* bone mineral density

Adjusted for birth weight, maternal smoking during pregnancy, gestational age, skin color, schooling (years - 18y), asset index (quintiles - 22y), smoking status (18 and 22y), alcohol use (AUDIT - 18 and 22y), physical activity (minutes per week 18 and 22y), medical diagnosis of asthma, diabetes and hypertension (22y), BMI (continuous - 22y), height (22y), daily calcium intake (18y and 22y), any kind of corticoids use in the last three months (22y), insulin (22y) and testosterone (22y)

*P*-values by Wald’s test for linear tendency, except ^a^Wald’s test for heterogeneity

**Table 4:** CRP and Il-6 at 18 and 22 years old and BMD at 22 years, females

	BMD - 22 years (mg/cm^2^)					
	Total body β (95% CI)		Lumbar spine β (95% CI)		Femur neck β (95% CI)	
	Crude	Adjusted	Crude	Adjusted	Crude	Adjusted
18 years						
CRP (log mg/L - tertiles)	*p < 0.001*	*p = 0.265*	*p < 0.001*	*p = 0.359*	*p < 0.001*	*p = 0.953*
1	Ref.	Ref.	Ref.	Ref.	Ref.	Ref.
2	18.7 (9.09; 28.3)	2.4 (−7.6; 11.2)	28.3 (12.8; 43.9)	9.8 (− 7.2; 26.7)	15.7 (0.1; 31.3)	−6.2 (−22.4; 9.9)
3	33.4 (23.8; 43.0)	5.8 (−4.4; 16.0)	40.1 (24.4; 55.7)	8.5 (−9.8; 26.8)	37.1 (21.4; 52.8)	0.5 (−17.0; 18.0)
IL-6 (log pg/mL - tertiles)	*p < 0.001*	*p = 0.506*	*p < 0.001*	*p = 0.217*	*p < 0.001*	*p = 0.730*
1	Ref.	Ref.	Ref.	Ref.	Ref.	Ref.
2	14.5 (4.7; 24.4)	2.2 (−6.8; 11.2)	27.7 (11.8; 43.7)	14.6 (−1.6; 30.8)	16.2 (0.2; 32.1)	1.2 (−14.2; 16.7)
3	28.3 (18.5; 38.2)	3.2 (−6.3; 12.7)	40.7 (24.7; 56.6)	10.3 (−6.7; 27.4)	39.4 (23.4; 55.3)	2.9 (−13.4; 19.1)
CRP and IL-6 in the highest tertile	*p < 0.001*	*p = 0.353*	*p < 0.001*	*p = 0.651*	*p < 0.001*	*p = 0.588*
None	Ref.	Ref.	Ref.	Ref.	Ref.	Ref.
Only CRP or IL-6	11.7 (2.8; 20.9)	0.7 (−7.9; 9.3)	13.0 (−1.8; 27.7)	−2.1 (− 17.5; 13.3)	15.8 (1.0; 30.5)	1.5 (− 13.2; 16.2)
Both CRP and IL-6	38.6 (27.4; 49.9)	5.9 (− 5.2; 16.9)	45.3 (27.1; 63.6)	6.5 (− 13.4; 26.3)	52.3 (34.1; 70.5)	5.4 (− 13.5; 24.5)
22 years						
CRP (log mg/L - tertiles)	*p < 0.001*	*p = 0.004*	*p < 0.001*	*p = 0.330*	*p < 0.001*	*p = 0.630*
1	Ref.	Ref.	Ref.	Ref.	Ref.	Ref.
2	18.6 (9.1; 28.1)	−3.3 (−12.6; 5.9)	25.0 (9.6; 40.4)	2.8 (−13.8; 19.4)	22.0 (6.6; 37.3)	−2.1 (− 17.9; 13.7)
3	24.9 (15.1; 34.6)	−15.2 (− 25.4; −4.9)	28.5 (12.7; 44.2)	−9.0 (− 27.4; 9.4)	35.5 (19.8; 51.2)	− 4.3 (− 21.9; 13.3)
IL-6 (log pg/mL - tertiles)	*p < 0.001*	*p = 0.133*	*p < 0.001*	*p = 0.813*	*p < 0.001*	*p = 0.163*
1	Ref.	Ref.	Ref.	Ref.	Ref.	Ref.
2	17.7 (7.9; 27.5)	−0.7 (−9.8; 8.3)	29.1 (13.2; 45.0)	10.6 (−5.6; 26.8)	29.9 (13.1; 44.8)	3.3 (−12.1; 18.8)
3	31.6 (21.7; 41.5)	−8.3 (−18.7; 2.1)	34.0 (17.9; 50.1)	−3.6 (−22.3; 15.1)	42.6 (26.5; 58.6)	−13.9 (−31.7; 4.0)
CRP and IL-6 in the highest tertile	*p < 0.001*	*p = 0.003*	*p = 0.003*	*p = 0.089*	*p < 0.001*	*p = 0.105*
None	Ref.	Ref.	Ref.	Ref.	Ref.	Ref.
Only CRP or IL-6	21.2 (11.9; 30.6)	−1.9 (−10.7; 6.9)	24.7 (9.5; 39.9)	−1.6 (− 17.5; 14.2)	24.8 (9.6; 40.0)	−5.0 (−20.2; 10.2)
Both CRP and IL-6	26.0 (14.9; 37.0)	−20.0 (−31.7; −8.3)	21.6 (3.7; 39.6)	− 20.0 (−41.2; 1.0)	36.4 (18.5; 54.3)	−17.5 (− 37.7; 2.8)
18 and 22 years^a^						
CRP in the highest tertile	*p < 0.001*	*p = 0.002*	*p = 0.002*	*p = 0.455*	*p < 0.001*	*p = 0.464*
None	Ref.	Ref.	Ref.	Ref.	Ref.	Ref.
Only at 18	24.3 (12.8; 35.9)	13.9 (3.0; 24.9)	25.1 (6.3; 43.9)	8.8 (−11.0; 28.5)	30.7 (11.9; 49.5)	12.9 (−6.0; 31.7)
Only at 22	12.2 (0.52; 23.8)	−6.8 (−18.0; 4.3)	11.3 (−7.6; 30.1)	−7.8 (−28.0; 12.3)	20.9 (2.1; 39.8)	2.7 (−16.5; 21.9)
Both 18 and 22 years	31.3 (19.8; 42.8)	−10.0 (−21.6; 1.5)	32.4 (13.7; 51.0)	−8.6 (−29.5; 12.2)	40.9 (22.3; 59.5)	−4.3 (− 24.2; 15.6)
IL-6 in the highest tertile	*p < 0.001*	*p = 0.306*	*p < 0.001*	*p = 0.428*	*p < 0.001*	*p = 0.232*
None	Ref.	Ref.	Ref.	Ref.	Ref.	Ref.
Only at 18	4.6 (−6.5; 15.7)	1.9 (−8.4; 12.2)	10.6 (−7.5; 28.7)	−2.3 (−20.8; 16.1)	12.2 (−5.6; 30.2)	3.5 (−14.0; 21.1)
Only at 22	6.1 (−5.1; 17.4)	−9.4 (−20.3; 1.5)	0.7 (−17.7; 19.1)	− 16.1 (−35.6; 3.4)	7.6 (− 10.7; 26.0)	−16.5 (− 35.2; 2.2)
Both 18 and 22 years	43.5 (31.8; 55.2)	−4.3 (− 16.9; 8.3)	45.8 (26.8; 64.9)	−1.7 (− 24.3; 21.0)	57.3 (38.3; 76.3)	−12.3 (− 34.0; 9.4)
Both CRP and IL-6 in the highest tertile	*p < 0.001*	*p = 0.001*	*p < 0.001*	*p = 0.170*	*p < 0.001*	*p = 0.081*
None	Ref.	Ref.	Ref.	Ref.	Ref.	Ref.
Only at 18	30.3 (17.1; 43.45)	11.6 (−0.3; 23.6)	40.9 (19.5; 62.3)	12.7 (−9.0; 34.3)	48.1 (26.8; 69.4)	16.7 (−4.0; 37.3)
Only at 22	11.0 (−1.7; 23.7)	−15.5 (−27.9; −3.2)	5.9 (− 14.7; 26.5)	−16.9 (− 39.2; 5.4)	24.5 (3.8; 45.1)	−5.6 (− 26.9; 15.7)
18 and 22 years	44.6 (27.7; 61.4)	−20.3 (− 38.0; − 2.5)	42.0 (14.6; 69.4)	− 16.6 (− 48.7; 15.5)	52.4 (25.1; 79.7)	− 27.3 (− 57.9; 3.3)

Total females sample *n* = 1661

*IL-6* interleukin-6, *CRP* C-reactive protein, *BMD* bone mineral density

Adjusted for birth weight, maternal smoking during pregnancy, gestational age, skin color, schooling (years - 18y), asset index (quintiles - 22y), smoking status (18 and 22y), alcohol use (AUDIT - 18 and 22y), physical activity (minutes per week 18 and 22y), medical diagnosis of asthma, diabetes and hypertension (22y), BMI (continuous - 22y), height (22y), daily calcium intake (18y and 22y), any kind of corticoids use in the last three months (22y), insulin (22y), testosterone (22y), age at menarche, oral contraceptive use in the last year (18 and 22y) and current breastfeeding (22y)

*P*-values by Wald’s test for linear tendency, except ^a^Wald’s test for heterogeneity

Association between adiponectin and BMD can be seen in Tables 5 and 6, for males and females, respectively. An inverse association was found between adiponectin and BMD in most of the unadjusted and adjusted analysis. For males (Table 5), statistical significant association was observed for adiponectin in the third tertile at 22 years and total body BMD (− 23.3 mg/cm^2^; 95% CI: -35.5; − 11.1; *p* < 001), lumbar spine BMD (− 22.5 mg/cm^2^; 95% CI: -42.9; − 2.2; p = 0.003) and femural neck (− 31.8 mg/cm^2^ 95% CI: -55.5; − 9.1). For females (Table 6), a borderline significance was found for adiponectin at 22 y and total body BMD (− 9.9 mg/cm^2^ 95% CI: -18.3; 0.6; *p* = 0.058) and for the third tertile of adiponectin and lumbar spine BMD (− 17.8 mg/cm^2^ 95% CI: -34.9; − 0.9; *p* = 0.033).

**Table 5:** Adiponectin at 18 and 22 years old and BMD at 22 years, males

	BMD - 22 years (mg/cm^2^)					
	Total body β (95% CI)		Lumbar spine β (95% CI)		Femur neck β (95% CI)	
	Crude	Adjusted	Crude	Adjusted	Crude	Adjusted
18 years						
Adiponectin (μg/mL - tertiles)	*p = 0.005*	*p = 0.166*	*p = 0.186*	*p = 0.244*	*p = 0.089*	*p = 0.315*
1	Ref.	Ref.	Ref.	Ref.	Ref.	Ref.
2	−0.7 (−48.0; 46.5)	−6.4 (−58.6; 45.8)	30.5 (−33.0; 94.1)	23.5 (−16.3; 108.4)	20.5 (− 61.4; 102.4)	−22.3 (− 121.8; 77.2)
3	− 69.2 (−116.1; − 22.4)	−39.5 (−93.8; 14.9)	−44.5 (− 107.1; 18.1)	−58.8 (− 146.2; 28.6)	−72.4 (− 153.1; 8.2)	−51.6 (− 154.1; 50.0)
22 years						
Adiponectin (μg/mL - tertiles)	*p < 0.001*	*p < 0.001*	*p = 0.001*	*p = 0.030*	*p < 0.001*	*p = 0.006*
1	Ref.	Ref.	Ref.	Ref.	Ref.	Ref.
2	−24.3 (−36.4; −12.1)	−7.5 (−19.4; 4.4)	−15.3 (− 33.1; 2.5)	−4.6 (− 24.3; 15.1)	−28.6 (− 49.3; − 7.9)	−4.0 (− 26.0; 18.1)
3	− 40.3 (− 52.5; − 28.1)	− 23.3 (− 35.5; − 11.1)	− 31.1 (− 49.0; − 13.3)	− 22.5 (− 42.9; − 2.2)	− 55.2 (− 75.9; − 34.5)	− 31.8 (− 55.5; − 9.1)
18 and 22 years^a^						
Adiponectin in the highest tertile	*p = 0.015*	*p = 0.371*	*p = 0.279*	*p = 0.384*	*p = 0.148*	*p = 0.825*
None	Ref.	Ref.	Ref.	Ref.	Ref.	Ref.
Only at 18	− 65.8 (− 126.3; − 5.3)	− 47.0 (− 120.6; 26.5)	− 55.1 (− 138.5; 28.3)	−80.7 (− 200.8; 39.4)	− 41.2 (− 146.1; 63.7)	−28.4 (− 169.3; 112.5)
Only at 22	− 28.2 (− 95.8; 39.3)	− 39.6 (− 119.8; 40.5)	−6.1 (− 99.3; 87.1)	− 29.7 (− 160.8; 101.3)	− 7.4 (− 124.6; 109.9)	− 8.4 (− 162.2; 145.3)
Both 18 and 22 years	− 71.8 (− 120.9; − 22.6)	−37.4 (− 95.9; 21.1)	− 56.7 (− 123.4; 9.9)	− 65.9 (− 161.1; 29.3)	−96.8 (− 180.6; − 13.0)	−51.6 (− 163.3; 60.1)

Total males sample 18 years *n* = 138 / 22 years *n* = 1568

*BMD* bone mineral density

Adjusted for birth weight, maternal smoking during pregnancy, gestational age, skin color, schooling (years - 18y), asset index (quintiles - 22y), smoking status (18 and 22y), alcohol use (AUDIT - 18 and 22y), physical activity (minutes per week 18 and 22y), medical diagnosis of asthma, diabetes and hypertension (22y), BMI (continuous - 22y), height (22y), daily calcium intake (18y and 22y), any kind of corticoids use in the last three months (22y), insulin (22y) and testosterone (22y)

*P*-values by Wald’s test for linear tendency, except ^a^Wald’s test for heterogeneity

**Table 6:** Adiponectin at 18 and 22 years old and BMD at 22 years, females

	BMD - 22 years (mg/cm^2^)					
	Total body β (95% CI)		Lumbar spine β (95% CI)		Femur neck β (95% CI)	
	Crude	Adjusted	Crude	Adjusted	Crude	Adjusted
18 years						
Adiponectin (μg/mL - tertiles)	*p = 0.031*	*p = 0.609*	*p = 0.093*	*p = 0.246*	*p = 0.077*	*p = 0.854*
1	Ref.	Ref.	Ref.	Ref.	Ref.	Ref.
2	−52.6 (−91.0; − 14.2)	−6.4 (− 49.5; 36.8)	−60.7 (− 114.9; − 6.5)	2.4 (− 66.9; 71.6)	−52.0 (− 113.1; 9.1)	5.4 (− 70.1; 80.9)
3	− 43.3 (− 82.0; − 4.7)	9.8 (− 33.3; 52.9)	− 47.1 (− 101.7; 7.4)	38.4 (− 30.7; 107.6)	− 55.7 (− 117.2; 5.7)	−6.0 (− 81.6; 69.5)
22 years						
Adiponectin (μg/mL - tertiles)	*p < 0.001*	*p = 0.058*	*p < 0.001*	*p = 0.033*	*p < 0.001*	*p = 0.258*
1	Ref.	Ref.	Ref.	Ref.	Ref.	Ref.
2	−19.0 (−28.5; − 9.5)	0.78 (− 8.4; 9.9)	−16.8 (− 32.2; − 1.4)	3.5 (− 12.8; 20.0)	−30.7 (− 46.1; − 15.4)	4.9 (− 10.8; 20.6)
3	−39.6 (− 49.1; − 30.1)	−9.9 (− 18.3; 0.6)	−50.5 (− 65.9; − 35.2)	− 17.8 (− 34.9; − 0.9)	− 56.9 (− 72.2; − 41.5)	− 8.9 (− 25.0; 7.3)
18 and 22 years^a^						
Adiponectin in the highest tertile	*p = 0.010*	*p = 0.688*	*p = 0.153*	*p = 0.521*	*p = 0.082*	*p = 0.806*
None	Ref.	Ref.	Ref.	Ref.	Ref.	Ref.
Only at 18	6.4 (− 40.6; 53.4)	19.9 (− 30.2; 70.0)	0.2 (− 67.1; 67.4)	41.3 (− 39.2; 121.9)	16.0 (− 58.5; 90.4)	16.3 (− 71.6; 104.2)
Only at 22	−54.0 (− 99.1; − 8.8)	− 15.2 (− 61.2; 30.8)	−57.4 (− 122.0; 7.2)	− 23.2 (− 97.1; 50.7)	−41.2 (− 112.7; 30.4)	−4.7 (− 85.1; 75.8)
Both 18 and 22 years	− 56.6 (− 98.7; − 14.4)	−2.3 (− 51.0; 46.5)	−53.0 (− 113.4; 7.4)	18.1 (− 60.3; 96.4)	− 77.1 (− 144.0; − 10.2)	− 34.3 (− 119.5; 51.0)

Total females sample 18 years *n* = 137 / 22 years *n* = 1688

*BMD* bone mineral density

Adjusted for birth weight, maternal smoking during pregnancy, gestational age, skin color, schooling (years - 18y), asset index (quintiles - 22y), smoking status (18 and 22y), alcohol use (AUDIT - 18 and 22y), physical activity (minutes per week 18 and 22y), medical diagnosis of asthma, diabetes and hypertension (22y), BMI (continuous - 22y), height (22y), daily calcium intake (18y and 22y), any kind of corticoids use in the last three months (22y), insulin (22y), testosterone (22y), age at menarche, oral contraceptive use in the last year (18 and 22y) and current breastfeeding (22y)

*P*-values by Wald’s test for linear tendency, except ^a^Wald’s test for heterogeneity

The findings using continuous exposures for linear regression, instead of tertiles, were the same for both analysis (Additional file 1: Table S1 and Additional file 2: Figures S1 to S6).

## Discussion

In general, our findings point to an inverse association mainly between CRP and adiponectin at 22 years and BMD at the same age, although most of the results did not reach statistical significance. The inverse association observed in the adjusted analysis was due to the presence of negative confounders; statistical significance was present for the association of higher tertiles of CRP at 22 y and total body BMD, in females. Higher tertiles of adiponectin at 22 years showed an inverse and significant association with BMD in the adjusted analysis for total body, lumbar spine and femoral neck BMD, in males, and for lumbar spine BMD, in females.

An important message in the present paper is the change of the direction of some regression coefficients between IL-6, CRP and BMD, after adjustment for confounders; the main confounder responsible for the change from positive to negative (or inverse) beta, was the covariate BMI. This can be seen in the Additional file 3: Table S2; there was a positive association between BMD and the exposures in the crude analysis, but the addition of all confounders in the full adjusted model changed the direction of the association from positive to negative regression coefficients; if we do not add BMI as a confounder, the direction of the association does not change. BMI is highly associated with IL-6 and CRP in a positive direction according to our previous paper on the same cohort [8]. Any analysis on the association of these cytokines and densitometry should always take into account adiposity, otherwise the results can be misleading. On the other hand, adiponectin is inversely associated with BMI [8], therefore, the inclusion of this covariate in the model did not change the direction of the association with BMD, with an inverse beta remaining in most of the unadjusted and adjusted analysis.

It is well known that the pubertal maturation affects bone size and its trajectory from birth to a maximal value by the end of the second or beginning of the third decade, according to the gender and skeletal site examined. Therefore, in young adults the sexual dimorphisms in BMD appears to be due to a greater gain in bone size in males than in females during puberty [19]; in our cohort the increase in the mean total body BMD in males from 18 to 22 years was 3.6% compared to 2.0% in females. In this way, adjustment for hormonal covariates should be performed; although testosterone, hormonal contraceptive use and age at menarche have been taken into account in our analysis, we were not able to adjust for blood female hormones, since they were not available in the cohort; we cannot exclude residual confounding as a possible explanation for the differences found according to sex and age in the present paper.

The literature shows some studies on cytokines and BMD, but most of these data have been obtained from small sample sizes, studies composed only by women, older ages and cross-sectional designs [20–22]. In the ALSPAC cohort the authors investigated the relationship between adiponectin at the age of 9.9 years and BMD at this age and at 15 years measured by DXA and tomography; IL-6 and CRP were considered as confounders in the analysis. The authors found an inverse effect size between adiponectin and BMD after adjustment for fat mass, height and lean mass and stronger associations in boys than in girls [7], similar to our findings. This raised the possibility that puberty partly attenuates the influence of adiponectin on bone development, particularly in girls. It has been postulated that rising estrogen levels influence cortical bone development in pubertal girls [23], although the basis for any interaction with adiponectin is currently unknown; puberty did not entirely weaken the relationship between adiponectin and bone mass observed in the ALSPAC cohort.

A systematic review and meta-analysis from Biver [24] on the influence of adipokines on bone mineral density concluded that adiponectin is the most relevant adipokine (among leptin, resistin and visfatin) negatively associated with BMD (pooled r from - 0.14 to - 0.4) independent of gender, fat mass parameters and menopausal status; it should be mentioned that most of the 59 studies included in this meta-analysis were cross-sectional and subjects aged less than 18 years were excluded. It has been proposed that adiponectin can activate transcription factor NF- κB regulating pro-inflammatory gene expression according to its oligomerization state [25]. In our young cohort we also found the highest inverse regression coefficients for adiponectin at 22 years and BMD (in males) compared to the other inflammatory markers. It should be mentioned that a possible reason for the lack of statistical significance at 18 years could be the small sample size at this age (we did not measure adiponectin in the whole cohort at the 18 y follow-up due to funding); although the higher negative regression coefficients for adiponectin and BMD at all sites, the confidence intervals were very large.

A recent review paper from Naot [26] mentioned three studies where circulating adiponectin was inversely correlated with BMD and positively correlated with an increased risk of fracture; this fracture risk was observed in men only [27–29], and the authors suggest that the effect of adiponectin on bones is sex-dependent, and that in men, adiponectin could be a novel risk factor for increased fracture risk, independent of body composition and BMD.

Limitations of the present study are related to the absence of potential confounders, such as: a) blood measures of progesterone and estrogen, which could explain the different findings between sexes; b) osteocalcin measurement as a marker of bone formation; and c) the performance of only DXA to assess BMD without tomography, which would allow us to assess the bone periosteal circumference and cortical thickness. Regarding the measurement of adiponectin, we can also mention that adiponectin exists as high-, medium-, and low-molecular-weight isoforms, which may have distinct functions; high-molecular-weight adiponectin may be a better predictor of insulin resistance and diabetes than total adiponectin [38], but in the present study we measured only total adiponectin levels; at 18 years, due to economic reasons, we were able to measure adiponectin in a very small sample. Non fasting blood can be also considered as a limitation, although in large health cohort studies as the present one it is very difficult to obtain fasting samples of blood. Another limitation of our study is the fact that we evaluated BMD at one point in time and we cannot be certain about the future implications of the biomarkers on the BMD.

The main strengths of this study are the longitudinal design, which allowed us to explore the exposure at two follow-ups (18 and 22 years); the adjustment for relevant confounders during the whole life-course from pregnancy to the beginning of adulthood; and the good response rates at the different follow-ups, ensuring the representativeness of the original sample.

## Conclusion

Taken together, our findings suggest that adiponectin at 22 years can be a marker for BMD at the same age, in males; CRP and adiponectin also showed a negative association with total body and lumbar spine BMD, respectively, in females; long-term implications of these cytokines as markers for skeletal strength still need to be more investigated in longitudinal studies.

## Additional files

Additional file 1: **Table S1.** Crude and adjusted linear regressions between continuous CRP, Il-6 and adiponectin at 18 and 22 years old and BMD at 22 years. (DOCX 79 kb)

Additional file 2: **Figure S1.** Interleukin-6 (IL-6) and C-reactive protein (CRP) at 18 years and bone mineral density (BMD - mg/cm^2^) at 22 years, males. **Figure S2.** Interleukin-6 (IL-6) and C-reactive protein (CRP) and bone mineral density (BMD - mg/cm^2^) at 22 years, males. **Figure S3.** Interleukin-6 (IL-6) and C-reactive protein (CRP) at 18 years and bone mineral density (BMD - mg/cm^2^) at 22 years, females. **Figure S4.** Interleukin-6 (IL-6) and C-reactive protein (CRP) and bone mineral density (BMD - mg/cm^2^) at 22 years, females. **Figure S5.** Adiponectin (μg/mL) at 18 and 22 years and bone mineral density (BMD - mg/cm^2^) at 22 years, males. **Figure S6.** Adiponectin (μg/mL) at 18 and 22 years and bone mineral density (BMD - mg/cm^2^) at 22 years, females. (PDF 1104 kb)

Additional file 3 **Table S2.** CRP and IL-6 at 18 and 22 years old and BMD at 22 years, females - models with and without BMI comparison. (DOCX 70 kb)

## Abbreviations

BMD: Bone mineral density
BMI: Body mass index
CI: Confidence interval
CRP: C-reactive protein
IL-6: Interleukin-6
SD: Standard deviation
SR: Soluble receptors
TNFα: Tumor necrosis factor alpha
