# Supplementary material for: Are cytokines (IL-6, CRP and adiponectin) associated with bone mineral density in a young adult birth cohort?
Source: BMC Musculoskelet Disord. 2018 Nov 30;19:427. doi: 10.1186/s12891-018-2357-3 (PMC6267914; doi:10.1186/s12891-018-2357-3)
Supplement: Supplementary file 1 — Table S1. Crude and adjusted linear regressions between continuous CRP, Il-6 and adiponectin at 18 and 22 years old and BMD at 22 years. (DOCX 79 kb) [file 12891_2018_2357_MOESM1_ESM.docx]

| Additional file 1: Table S1. Crude and adjusted linear regressions between continuous CRP, Il-6 and adiponectin at 18 and 22 years old and BMD at 22 years. | | | | | | |
| --- | --- | --- | --- | --- | --- | --- |
|  | **BMD - 22 years (mg/cm²)** | | | | | |
|  | **Total body**  **β (95% CI)** | | **Lumbar spine**  **β (95% CI)** | | **Femur neck**  **β (95% CI)** | |
|  | **Crude** | **Adjusted** | **Crude** | **Adjusted** | **Crude** | **Adjusted** |
| **MALES** |  |  |  |  |  |  |
| **18 years** |  |  |  |  |  |  |
| CRP (log mg/L) | p= 0.172  6.9 (-3.0; 16.7) | p= 0.371  -4.3 (-13.8; 5.2) | p= 0.313  7.4 (-7.0; 21.7) | p= 0.550  -4.8 (-20.6; 10.9) | p= 0.688  3.4 (-13.3; 20.2) | p= 0.407  -7.4 (-25.0; 10.1) |
| IL-6 (log pg/mL) | p= 0.661  -2.2 (-12.2; 7.7) | p= 0.046  -9.9 (-19.7; -0.2) | p= 0.501  -5.0 (-19.5; 9.5) | p= 0.147  -11.8 (-27.8; 4.2) | p= 0.847  -1.7 (-18.6; 15.3) | p= 0.279  -9.9 (-27.9; 8.1) |
| Adiponectin (µg/mL) | p= 0.009  -4.1 (-7.2; -1.0) | p= 0.336  -1.7 (-5.2; 1.8) | p= 0.175  -2.8 (-7.0; 1.3) | p= 0.398  -2.4 (-8.0; 3.2) | p= 0.075  -4.8 (-10.1; 0.5) | p= 0.565  -2.0 (-8.7; 4.8) |
| **22 years** |  |  |  |  |  |  |
| CRP (log mg/L) | p= 0.021  10.9 (1.6; 20.1) | p= 0.939  -0.4 (-9.5; 8.8) | p= 0.070  12.5 (-1.0; 26.1) | p= 0.192  10.1 (-5.1; 25.3) | p= 0.042  16.4 (0.6; 32.1) | p= 0.755  2.7 (-14.3; 19.7) |
| IL-6 (log pg/mL) | p< 0.001  17.9 (8.1; 27.8) | p= 0.234  6.0 (-3.9; 15.8) | p= 0.045  15.0 (0.4; 29.7) | p= 0.126  12.8 (-3.6; 29.2) | p= 0.009  22.6 (5.5; 39.6) | p= 0.181  12.5 (-5.8; 30.9) |
| Adiponectin (µg/mL) | p< 0.001  -5.3 (-6.9; -3.6) | p< 0.001  -3.3 (-4.9; -1.7) | p< 0.001  -4.6 (-7.0; -2.2) | p= 0.004  -4.0 (-6.7; -1.2) | p< 0.001  -6.9 (-9.8; -4.2) | p= 0.003  -4.6 (-7.6; -1.6) |
| **FEMALES** |  |  |  |  |  |  |
| **18 years** |  |  |  |  |  |  |
| CRP (log mg/L) | p< 0.001  14.8 (9.8; 19.7) | p= 0.346  2.5 (-2.7; 7.6) | p< 0.001  17.8 (9.8; 25.8) | p= 0.462  3.4 (-5.7; 12.5) | p< 0.001  16.0 (8.0; 24.0) | p= 0.773  1.3 (-7.5; 10.0) |
| IL-6 (log pg/mL) | p< 0.001  22.7 (15.2; 30.2) | p= 0.666  1.6 (-5.6; 8.9) | p< 0.001  33.3 (21.1; 45.5) | p= 0.160  9.4 (-3.7; 22.5) | p< 0.001  29.4 (17.3; 41.5) | p= 0.775  -1.8 (-14.2; 10.6) |
| Adiponectin (µg/mL) | p= 0.001  -4.1 (-6.5; -1.7) | p= 0.489  -1.0 (-3.9; 1.9) | p= 0.011  -4.4 (-7.9; -1.0) | p= 0.698  0.9 (-3.8; 5.7) | p= 0.003  -5.8 (-9.6; -2.0) | p= 0.301  -2.7 (-7.9; 2.5) |
| **22 years** |  |  |  |  |  |  |
| CRP (log mg/L) | p< 0.001  11.4 (6.7; 16.0) | p< 0.001  -9.3 (-14.3; -4.4) | p= 0.002  10.0 (2.5; 17.5) | p= 0.034  -9.6 (-18.4; -0.7) | p< 0.001  15.7 (8.2; 23.1) | p= 0.225  -5.2 (-13.7; 3.2) |
| IL-6 (log pg/mL) | p< 0.001  24.2 (17.0; 31.4) | p= 0.043  -7.9 (-15.5; -0.3) | p< 0.001  27.5 (15.9; 39.2) | p= 0.751  -2.2 (-16.0; 11.5) | p< 0.001  32.3 (20.6; 44.0) | p= 0.039  -13.9 (-27.1; -0.7) |
| Adiponectin (µg/mL) | p< 0.001  -4.2 (-5.2; -3.3) | p= 0.081  -0.9 (-1.9; 0.1) | p< 0.001  -5.2 (-6.9; -3.6) | p= 0.057  -1.7 (-3.5; 0.0) | p< 0.001  -6.0 (-7.6; -4.3) | p= 0.372  -0.8 (-2.5; 0.9) |

Total males sample n= 1486. Total females sample n= 1661.

Adiponectin 18y females n= 137/ males n= 138

IL-6: interleukin-6; CRP: C-reactive protein; BMD: bone mineral density.

Males: adjusted for birth weight, maternal smoking during pregnancy, gestational age, skin color, schooling (years - 18y), asset index (quintiles - 22y), smoking status (18 and 22y), alcohol use (AUDIT - 18 and 22y), physical activity (minutes per week 18 and 22y), medical diagnosis of asthma, diabetes and hypertension (22y), BMI (continuous - 22y), height (22y), daily calcium intake (18y and 22y), any kind of corticoids use in the last three months (22y), insulin (22y) and testosterone (22y). Females: same model + age at menarche, oral contraceptive use in the last year (18 and 22y) and current breastfeeding (22y).

P-values by Wald’s test for linear tendency.
