# Supplementary material for: Are cytokines (IL-6, CRP and adiponectin) associated with bone mineral density in a young adult birth cohort?
Source: BMC Musculoskelet Disord. 2018 Nov 30;19:427. doi: 10.1186/s12891-018-2357-3 (PMC6267914; doi:10.1186/s12891-018-2357-3)
Supplement: Supplementary file 2 — Figure S1. Interleukin-6 (IL-6) and C-reactive protein (CRP) at 18 years and bone mineral density (BMD - mg/cm2) at 22 years, males. Figure S2. Interleukin-6 (IL-6) and C-reactive protein (CRP) and bone mineral density (BMD - mg/cm2) at 22 years, males. Figure S3. Interleukin-6 (IL-6) and C-reactive protein (CRP) at 18 years and bone mineral density (BMD - mg/cm2) at 22 years, females. Figure S4. Interleukin-6 (IL-6) and C-reactive protein (CRP) and bone mineral density (BMD - mg/cm2) at 22 years, females. Figure S5. Adiponectin (μg/mL) at 18 and 22 years and bone mineral density (BMD - mg/cm2) at 22 years, males. Figure S6. Adiponectin (μg/mL) at 18 and 22 years and bone mineral density (BMD - mg/cm2) at 22 years, females. (PDF 1104 kb) [file 12891_2018_2357_MOESM2_ESM.pdf]

Supplementary material.

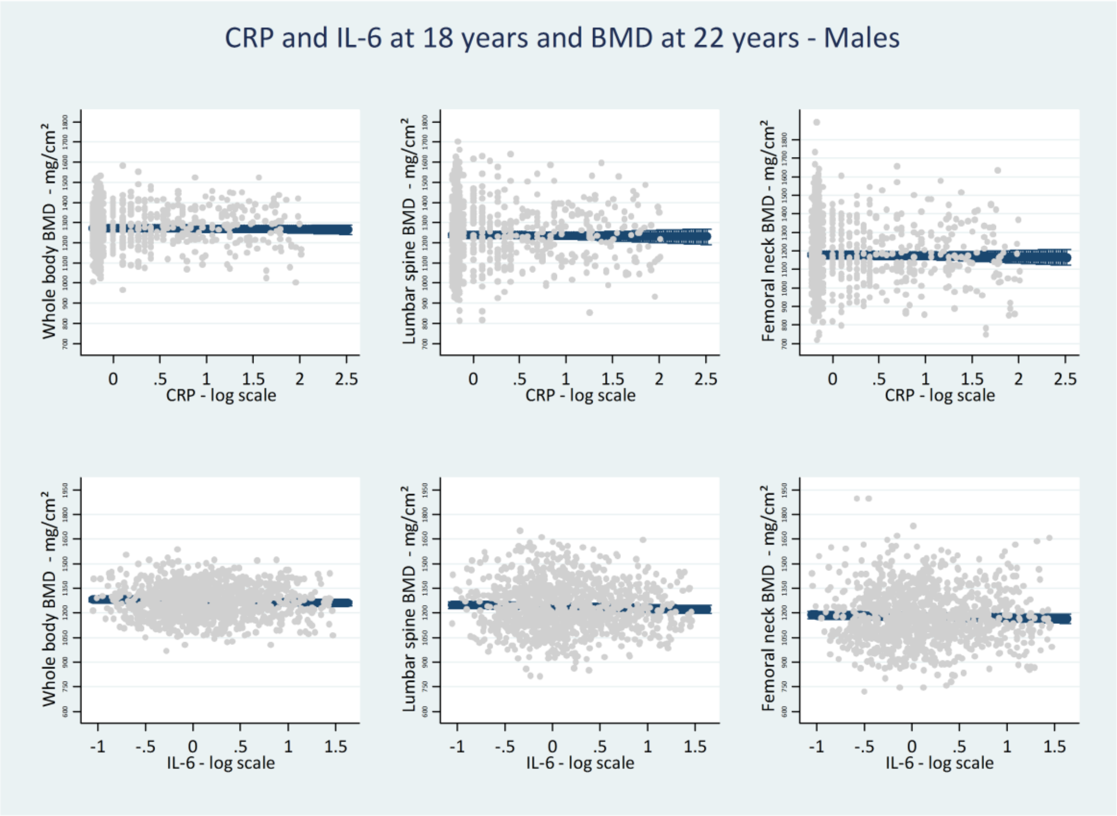

Supplementary Figure 1. Interleukin-6 (IL-6) and C-reactive protein (CRP) at 18 years and bone mineral density (BMD - mg/cm²) at 22 years, males.

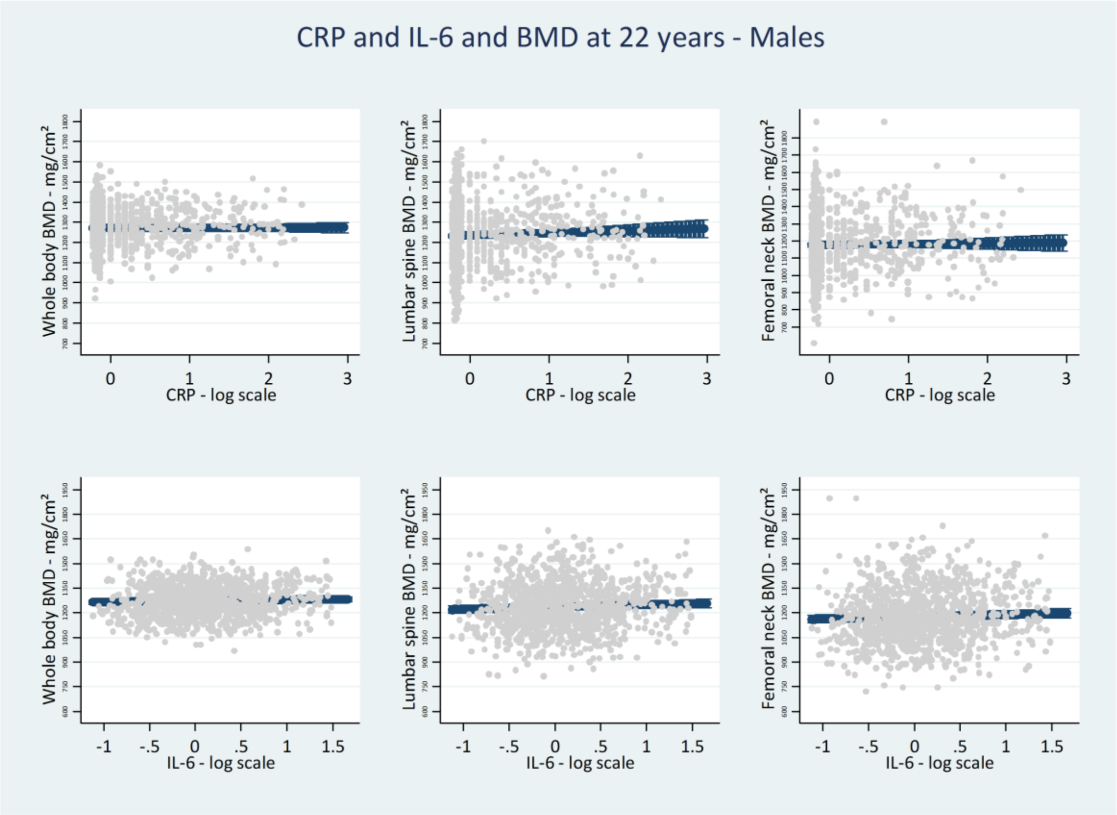

Supplementary Figure 2. Interleukin-6 (IL-6) and C-reactive protein (CRP) and bone mineral density (BMD - mg/cm²) at 22 years, males.

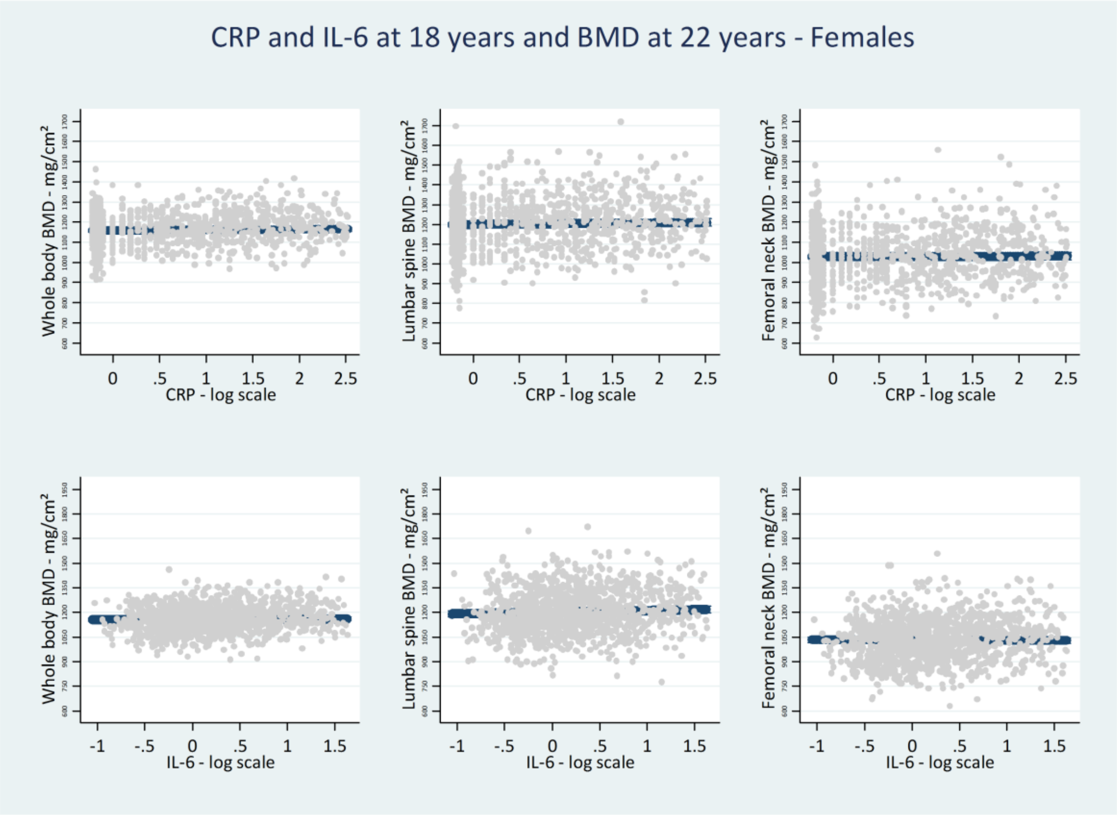

Supplementary Figure 3. Interleukin-6 (IL-6) and C-reactive protein (CRP) at 18 years and bone mineral density (BMD - mg/cm²) at 22 years, females.

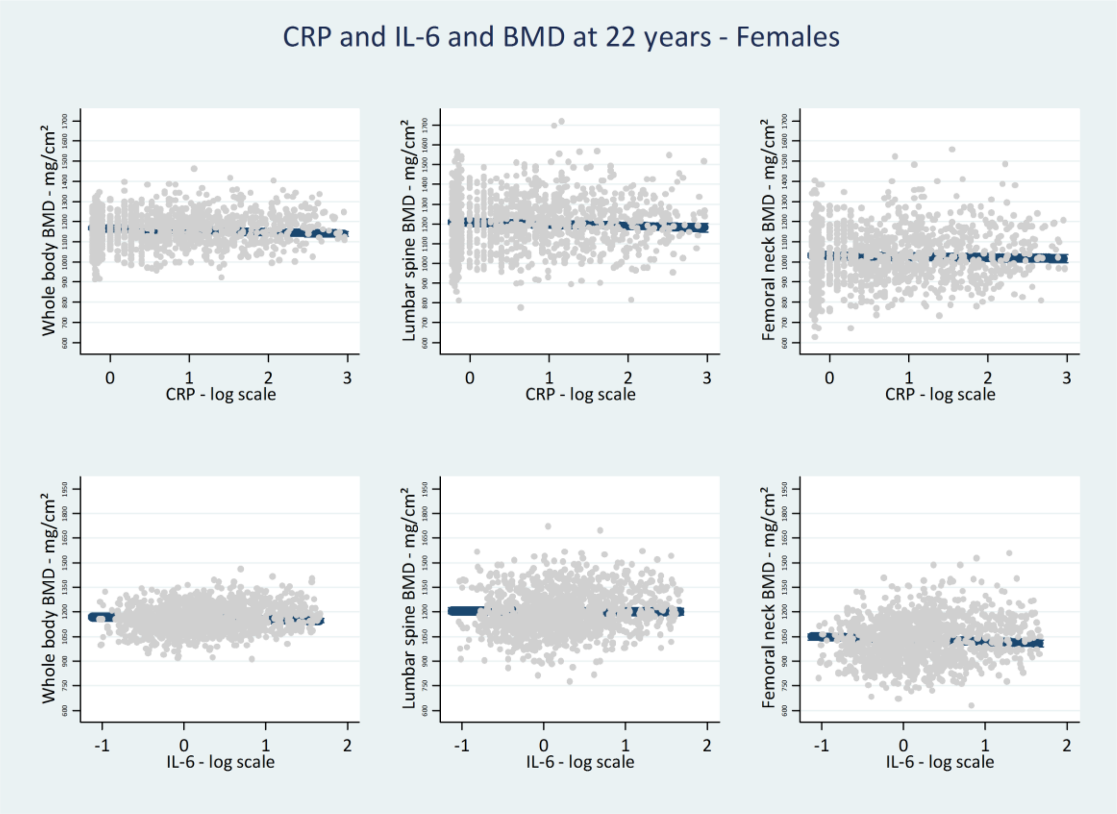

Supplementary Figure 4. Interleukin-6 (IL-6) and C-reactive protein (CRP) and bone mineral density (BMD - mg/cm²) at 22 years, females.

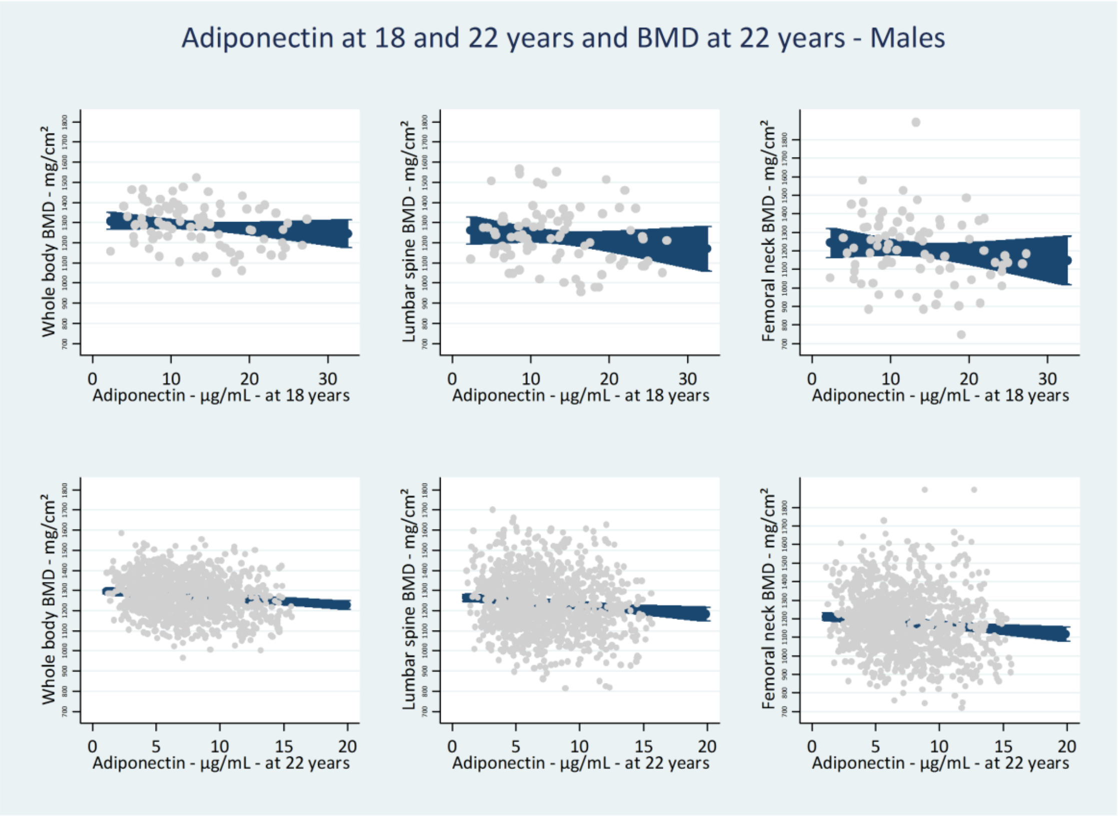

Supplementary Figure 5. Adiponectin (µg/mL) at 18 and 22 years and bone mineral density (BMD - mg/cm²) at 22 years, males.

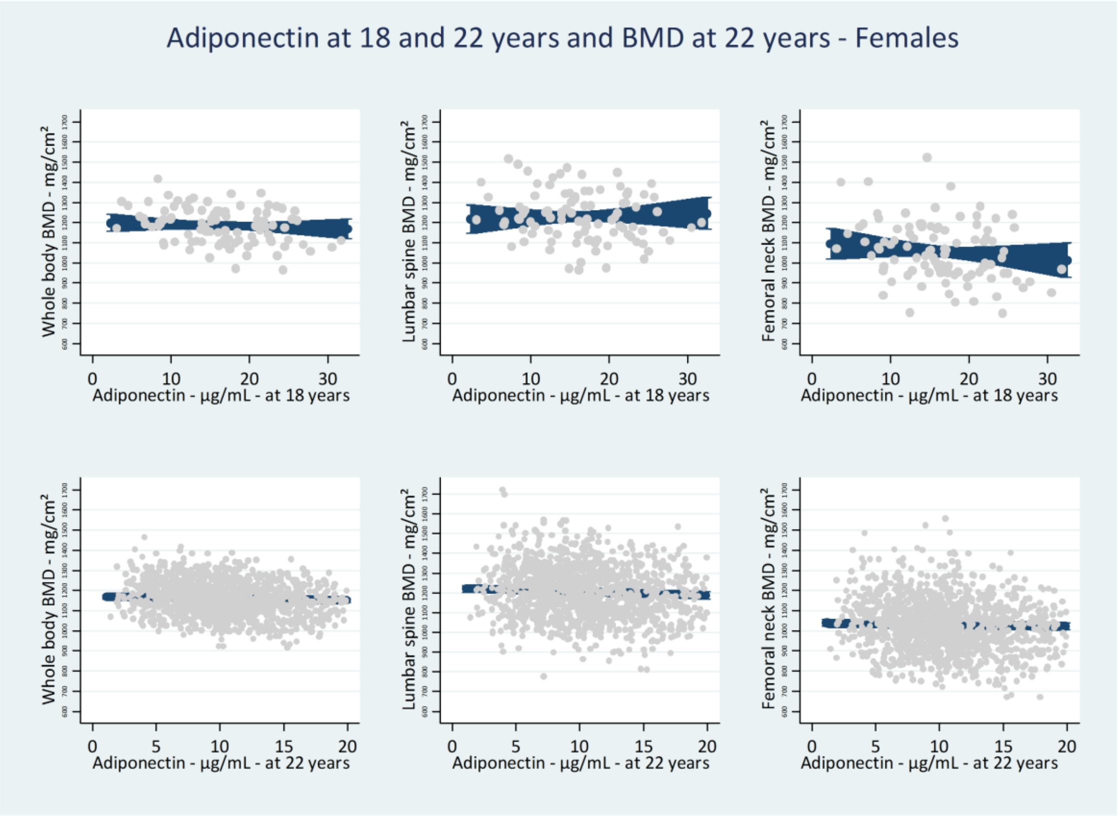

Supplementary Figure 6. Adiponectin (µg/mL) at 18 and 22 years and bone mineral density (BMD - mg/cm²) at 22 years, females.
