# Supplementary material for: Are cytokines (IL-6, CRP and adiponectin) associated with bone mineral density in a young adult birth cohort?
Source: BMC Musculoskelet Disord. 2018 Nov 30;19:427. doi: 10.1186/s12891-018-2357-3 (PMC6267914; doi:10.1186/s12891-018-2357-3)
Supplement: Supplementary file 3 — Table S2. CRP and IL-6 at 18 and 22 years old and BMD at 22 years, females - models with and without BMI comparison. (DOCX 70 kb) [file 12891_2018_2357_MOESM3_ESM.docx]

|  | **Total body BMD - 22 years (mg/cm²)** | | |
| --- | --- | --- | --- |
| **22 years** | **Crude** | **Adjusted full model** | **Adjusted without BMI** |
| **CRP (log mg/L - tertiles)** | *p< 0.001* | *p=* *0.004* | *p< 0.001* |
| 1 | Ref. | Ref. | Ref. |
| 2 | 18.6 (9.1; 28.1) | -3.3 (-12.6; 5.9) | 16.4 (6.2; 26.7) |
| 3 | 24.9 (15.1; 34.6) | -15.2 (-25.4; -4.9) | 26.4 (15.7; 37.2) |
| **CRP and IL-6 in the highest tertile** | *p< 0.001* | *p= 0.003* | *p< 0.001* |
| None | Ref. | Ref. | Ref. |
| Only CRP or IL-6 | 21.2 (11.9; 30.6) | -1.9 (-10.7; 6.9) | 20.7 (11.0; 30.4) |
| Both CRP and IL-6 | 26.0 (14.9; 37.0) | -20.0 (-31.7; -8.3) | 31.9 (20.1; 43.8) |
| **18 and 22 years** |  |  |  |
| **Both CRP and IL-6 in the highest tertile*** | *p< 0.001* | *p=*  *0.001* | *p< 0.001* |
| None | Ref. | Ref. | Ref. |
| Only at 18 | 30.3 (17.1; 43.45) | 11.6 (-0.3; 23.6) | 26.6 (13.2; 40.1) |
| Only at 22 | 11.0 (-1.7; 23.7) | -15.5 (-27.9; -3.2) | 17.7 (4.3; 31.2) |
| 18 and 22 years | 44.6 (27.7; 61.4) | -20.3 (-38.0; -2.5) | 49.4 (30.9; 67.8) |

Additional file 3: Table S2 CRP and IL-6 at 18 and 22 years old and BMD at 22 years, females - models with and without BMI comparison

Total females sample n= 1661.

IL-6: interleukin-6; CRP: C-reactive protein; BMD: bone mineral density.

Adjusted for birth weight, maternal smoking during pregnancy, gestational age, skin color, schooling (years - 18y), asset index (quintiles - 22y), smoking status (18 and 22y), alcohol use (AUDIT - 18 and 22y), physical activity (minutes per week 18 and 22y), medical diagnosis of asthma, diabetes and hypertension (22y), BMI (continuous - 22y), height (22y), daily calcium intake (18y and 22y), any kind of corticoids use in the last three months (22y), insulin (22y), testosterone (22y), age at menarche, oral contraceptive use in the last year (18 and 22y) and current breastfeeding (22y).

P-values by Wald’s test for linear tendency, except *Wald’s test for heterogeneity.
